# Oil supplementation with a special combination of n-3 and n-6 long-chain polyunsaturated fatty acids does not protect for exercise induced asthma: a double-blind placebo-controlled trial

**DOI:** 10.1186/s12944-020-01343-2

**Published:** 2020-07-13

**Authors:** M. Dreßler, D. Fussbroich, L. Böhler, E. Herrmann, N. Benker, M. Tytyk, J. Schulze, R. Schubert, C. Beermann, S. Zielen

**Affiliations:** 1 Department for Children and Adolescents, Division of Allergology, Pulmonology and Cystic fibrosis, Goethe-University, Frankfurt/Main, Germany; 2 Department of Food Technology, University of Applied Science, Fulda, Germany; 3 Faculty of Biological Sciences, Goethe-University, Frankfurt/Main, Germany; 4 Institute of Biostatistics and Mathematical Modelling, Goethe-University, Frankfurt/Main, Germany

**Keywords:** Exercise-induced asthma, Exercise challenge, Forced expiratory volume in 1 s, Exhaled nitric oxide, Long-chain polyunsaturated fatty acids, Double-blind placebo-controlled trial

## Abstract

**Background:**

Many patients suffering from exercise-induced asthma (EIA) have normal lung function at rest and show symptoms and a decline in FEV_1_ when they do sports or during exercise-challenge. It has been described that long-chain polyunsaturated fatty acids (LCPUFA) could exert a protective effect on EIA.

**Methods:**

In this study the protective effect of supplementation with a special combination of n-3 and n-6 LCPUFA (sc-LCPUFA) (total 1.19 g/ day) were investigated in an EIA cold air provocation model. Primary outcome measure: Decrease in FEV_1_ after exercise challenge and secondary outcome measure: anti-inflammatory effects monitored by exhaled NO (eNO) before and after sc-LCPUFA supplementation versus placebo.

**Results:**

Ninety-nine patients with exercise-induced symptoms aged 10 to 45 were screened by a standardized exercise challenge in a cold air chamber at 4 °C. Seventy-three patients fulfilled the inclusion criteria of a FEV_1_ decrease > 15% and were treated double-blind placebo-controlled for 4 weeks either with sc-LCPUFA or placebo. Thirty-two patients in each group completed the study. Mean FEV_1_ decrease after cold air exercise challenge and eNO were unchanged after 4 weeks sc-LCPUFA supplementation.

**Conclusion:**

Supplementation with sc-LCPUFA at a dose of 1.19 g/d did not have any broncho-protective and anti-inflammatory effects on EIA.

**Trial registration:**

Clinical trial registration number: NCT02410096***.*** Registered 7 February 2015 at Clinicaltrial.gov

## Introduction

Bronchial asthma is one of the most common chronic diseases and has considerable economic and health relevance [1]. Asthma triggers include respiratory infections, allergens, airborne pollutants and physical stress. Many patients suffering from exercise induced asthma (EIA) have normal lung function at rest but show symptoms and a decline in FEV_1_ of at least 10–15% during exercise-challenge. EIA is particularly prevalent among children and adolescents.

Exhaled NO (eNO) as marker of eosinophil airway inflammation has been described as a good predictor of EIA, and several studies have shown higher eNO levels among subjects with EIA [2–7].

Although pharmacological treatment is well established, alternative treatment options become more and more important for the patients in order to reduce their daily amount of pharmacologic medication. The first step of non-pharmacologic treatment of EIA involves advising the patient to warm up slowly and to avoid exercising in cold weather or on high-pollen days [8]. In addition, long-chain polyunsaturated fatty acids (LCPUFA) have been suggested as a possible complementary / alternative therapy for EIA. Especially n-3 long-chain PUFA (n-3 LCPUFA) exert well known anti-inflammatory effects, which are in part associated with a change in cell membrane composition [8, 9].

In line with this concept many studies demonstrated that supplementation of n-3 LCPUFA have beneficial effects on EIA [10–15]. In contrast, there are many studies which failed to show clinical improvement of EIA by n-3 LCPUFA supplementation [16–22]. The diverging study results on n-3 LCPUFA supplementation may be attributable to many factors, of which the poor design of earlier studies, i.e. small patient cohorts and different concentration and duration of n-3 LCPUFA supplementation are likely affecting the results. Therefore, a synergistic combination of n-3 and n-6 LCPUFA (sc-LCPUFA) containing eicosapentaenoic acid (EPA), docosahexaenoic acid (DHA), echium seed oil containing stearidonic (SDA) and gamma-linolenic acid (GLA) was designed as recently described in a review [23]. The idea of this study was to test possible synergistic effects of a combination of these four different n-3 and n-6 PUFA species. SDA, GLA, EPA and DHA influence several immunologic parameters, connected to clinical aspects of the asthmatic inflammation addressing distinctively different metabolic and regulatory pathways [23]. This specific sc-LCPUFA is a promising approach to reduce eosinophilic inflammation and improve lung function in patients with asthma by restoring fatty acid homeostasis and increasing EPA and DHA levels as recently shown in an asthma mouse model [23, 24]. The supplementation of 1190 mg PUFA/d allows comparisons with other trials addressing PUFA-treatments of bronchial inflammation merely with EPA or DHA.

Taken together, the aim of this double-blind placebo-controlled study was to evaluate the effect of a sc-LCPUFA combination on the bronchoconstriction response during exercise provocation and on airway inflammation in a large sample size of patients suffering from EIA.

## Material and methods

### Study design

The double-blind placebo-controlled trial took place from April 2015 until January 2016 and consisted of 3 visits in a time frame of 4 to 8 weeks. Participation in the study was voluntary. Prior to the commencement of the first visit (Screening visit, V1), written informed consent was required from each subject or from the parents of children under the age of 18 years.

At V1, the participants’ inclusion and exclusion criteria were confirmed, a lung function test administered using the MasterScreen spirometer, a skin prick test (SPT) performed, eNO measured and an asthma control questionnaire (ACQ) provided to participants.

**Fig. 1:**
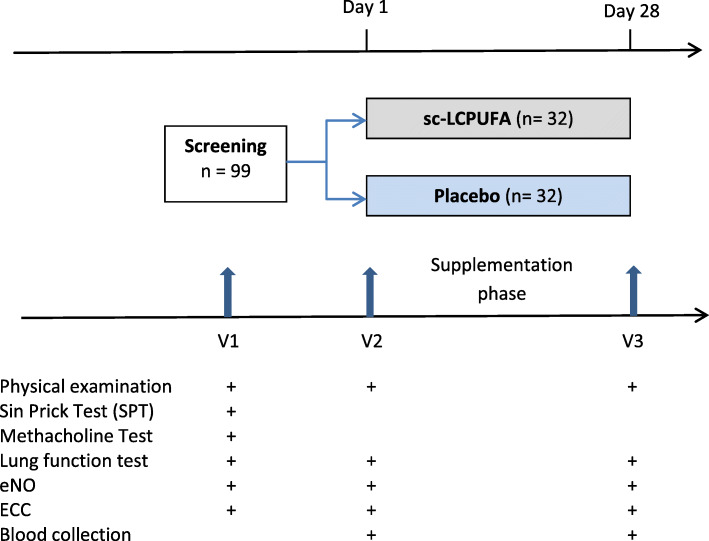
Flowchart of study design with assessments

On the second (V2, Randomization) and third visit (V3, End of study) a lung function test was conducted, eNO measured, an exercise challenge in a cold chamber (ECC) administered and blood collected. If the patient fulfilled the inclusion criteria `FEV_1_ decrease ≥15% in the ECC` at V2, they were randomised to one of the two study arms (interventional group with sc-LCPUFA supplementation or placebo group). V3 was performed after 4 weeks of supplementation. The study flow chart with assessments is shown in Fig.1. The study was approved by the ethics committee of Goethe University (reference number 360/14) Clinical trials registration number: NCT02410096.

### Subjects

Ninety-nine subjects aged 10 to 45 years with asthmatic symptoms while exercising were recruited from the outpatient clinic of the Department for Children and Adolescents, Division of Allergology, Pulmonology and Cystic fibrosis, Goethe-University, Frankfurt/Mail, Germany by a public posting. Subjects with chronic asthma and regular use of inhaled corticosteroids (ICSs) or leukotriene receptor antagonists were excluded. Further exclusion criteria were: subjects with FVC < 75%, subjects > 18 years/ < 18 years with FEV_1_ < 60/ < 70%, oral corticosteroids, other known chronic disease or infection, pregnancy, documented alcohol, medication or drug abuse and inability to perform and understand all study procedures.

### Sc-LCPUFA supplementation and placebo

The total amount of functional LCPUFA in the investigational product came to 1190 mg/day (710 mg EPA, 161 mg DHA, 175 mg GLA and 144 mg SDA). Therefore, the patients had to take 4 different capsules each morning combined with their breakfast: 1 capsule PlusEPA (containing EPA, Minami Nutrition, Aartselaar, Belgium); 1 capsule EPA/DHA/GLA (containing EPA/DHA/GLA, Peak Performance Products S.A., Grevenmacher, Luxemburg); 2 capsules Echiomega (containing SDA/GLA, Igennus Healthcare Nutrition, Cambridge, UK). The placebo compound (Allcura, naturheilmittel GmbH) was composed of 500 mg olive oil.

### Pulmonary function test

Baseline pulmonary function tests were performed using the MasterScreen spirometer (CareFusion, Germany). The following parameters were recorded: forced vital capacity (FVC), FEV_1_ and FEV_1_/FVC.

### Measurement of exhaled nitric oxide

Measurements of eNO were conducted using the NIOX1 gas analyzer (Aerocrine, Sweden). NIOX1 measures eNO in exhaled air according to ATS guidelines [25].

### Exercise challenge in a cold chamber

The exercise challenge was performed according to the ATS guidelines for EIA [26] as recently described [7, 27]. The subjects ran in a cold chamber (Ilkazell Isoliertechnik, Germany) cooled to 2–4 °C (microprocessor-based controller, Dixell, Emerson Climate Technologies, United Kingdom) on a treadmill (Schiller Intertrack 8100 T Med, Germany) with an incline of 10% for 6 mins (≤ 12 years) or 8 mins (> 12 years). At 5, 10, 15 and 30 mins after running, spirometry were performed. A positive reaction was defined as a decline in FEV_1_ ≥ 15% from the baseline value at two points in time after exercise.

### Asthma control questionnaire (ACQ)

The ACQ has strong evaluative and discriminative properties and can be used with confidence to measure asthma control [28]. The ACQ assesses seven items, which include asking patients to recall their experiences in the previous week and to respond to questions about nighttime waking, symptoms on waking, activity limitations, shortness of breath, wheezing, required use of short-acting β_2_-agonists for rescue, and FEV_1_ percent predicted before bronchodilator on a 7-point scale [28].

The items were equally weighted and the ACQ score was the mean of the 7 items and therefore between 0 (totally controlled) and 6 (severely uncontrolled). Thus, patients with an ACQ < 0.75 have a good asthma control, patients with ACQ > 1.5 have an uncontrolled asthma, in-between the asthma is partially controlled [29, 30]. The ACQ is valid for use in children from the age of 6 [31].

### Verification of PUFA incorporation by gas-chromatography

The fatty acid profile of blood plasma and blood cells were separately determined by fatty acid methyl esters (FAME) analysis as previously described [24]. The FAMEs of plasma and blood cells were analyzed by capillary gas chromatography (CGC) (Trace1300, Thermo Scientific, Dreieich, Germany) equipped with an autosampler AS1310 (Thermo Fisher Scientific, Dreieich, Germany). Fatty acid separation was achieved by using a capillary pre-column GuardGOLD, length: 2 m, i.d.: 0.25 mm (Thermo Fisher Scientific, Milan, Italy) and downstream a capillary column TRACE TR-FAME (70% cyanopropyl polysilphenylene siloxane), length: 60 m, i.d.: 0.25 mm, film thickness: 0.25 μm (Thermo Fisher Scientific, Dreieich, Germany).

The gas chromatographic conditions were the following: injector (SSL): 250 °C, splitless and carrier gas: helium (purification 99%) at a flow of 20 mL x min^− 1^. Compounds were monitored by flame-ionization detector (FID) at 250 °C. Fatty acids were identified by comparison of their retention times with those of external standard (Supelco 37-Component FAME Mix, Sigma-Aldrich, St. Louis, Missouri, United States). The column oven temperature was maintained at 60 °C for 0.5 min after injection and then programmed at 40 min to 180 °C (held for 2 min), then at 2 min^− 1^ to 210 °C (held for 3 min) and finally at 3 min^− 1^ to 240 °C (held for 10 min). The total run-time was 44 min.

### Statistical analyses

GraphPad Prism 5.01 (GraphPad Software Inc., La Jolla, CA, USA) and R 3.6 (R Foundation for Statistical Computing, Vienna, Austria) were used for statistical analyses. Data are presented as median and range or as mean and standard deviation (SD).

The primary outcome measure was the decrease in FEV_1_ after exercise challenge in cold air as measured by spirometry (FEV_1_, percent predicted). The secondary outcomes were the anti-inflammatory effects monitored by exhaled NO (eNO) before and after PUFA supplementation versus placebo, the incorporation of the different components of the sc-LCPUFA supplementation and the side effects of the sc-LCPUFA supplementation.

Intra-group and inter-group comparisons were calculated either by One-Way ANOVA, paired t-test or Wilcoxon test and unpaired t-test or Mann-Whitney test, respectively, according to the Kolmogorov-Smirnov test for normal distribution. *P* < 0.05 was considered as statistically significant. We also compared the results with a more complex multivariable linear mixed effect analysis, especially for potential interactions.

Power Calculation: For the sample size calculation the results of a previous study were used: “Predictors and reproducibility of exercise-induced bronchoconstriction in cold air” [27]. Assuming an improvement of the maximal FEV_1_ decrease after ECC of 35% in the interventional group and a test power of 80% (corresponding to a probability of beta-error of β = 0.2) 30 patients in the interventional and placebo group for an effect with an alpha coefficient of 0.05 had to be investigated.

## Results

### Patient characteristics

Ninety nine patients with exercise-induced symptoms aged 10 to 45 were recruited for V1, 73 of them fulfilled all inclusion criteria and were randomized for the study and treated placebo-controlled for 4 weeks. With 9 drop-outs between V2 and V3, 64 patients (32 patients in each group) completed the study and entered the statistical calculations.

Fifty subjects (78.1%) showed a positive SPT and 32 (50.0%) suffered from symptoms of allergic rhinoconjunctivitis and were challenged beyond the pollen season. Twenty-four subjects (37.5%) suffered from cough during the pollen season, 48 (75.0%) had doctor’s diagnosed bronchial asthma and 26 of them (40.6%) used short-acting β_2_-agonists on demand. Thirty-three subjects (51.6%) showed an ACQ < 0.75, 18 (28.1%) an ACQ 0.75–1.5 and 13 (20.3%) an ACQ > 1.5. For the FEV_1_ and FVC measures, the ATS/European Respiratory Society test criteria for acceptability and repeatability were met [32]. The characteristics of the subjects are summarized in Table 1. There was no significant difference between the subgroups interventional and placebo for age, sex, BMI, FEV_1_, eNO and ACQ.

**Table 1:** Patient characteristics

		Total	Adult	Children	Placebo	sc-LCPUFA
**Subjects**	[n]	64	34	30	32	32
**Female/Male**	[n]	35/29	22/12	13/17	16/16	19/13
**Age**	[yrs]	19.0 ± 5.5	23.5 ± 3.0	13.8 ± 2.2	18.4 ± 5.0	19.5 ± 6.0
**Weight**	[kg]	61.0 ± 14.0	67.6 ± 12.4	53.6 ± 11.9	60.1 ± 13.6	62.0 ± 14.6
**Height**	[m]	1.7 ± 0.1	1.7 ± 0.1	1.6 ± 0.1	1.7 ± 0.1	1.7 ± 0.1
**FVC**	[% pred]	96.6 (70.2–140.1)	94.0 (70.2–117.3)	98.8 (77.7–140.1)	96.7 (70.2–114.7)	96.3 (82.6–140.1)
**FEV**_**1**_	[% pred]	94.9 (64.4–118.4)	91.8 (64.4–115.2)	97.3 (69.6–118.4)	95.3 (64.4–111.0)	92.9 (72.6–118.4)
**eNO**	[ppb]	27.5 (5.0–197.0)	27.5 (5.0–197.0)	28.5 (7.0–88.0)	31.5 (6.0–96.0)	20.5 (5.0–197.0)
**ACQ**		0.7 (0.0–2.9)	0.7 (0.0–2.9)	0.8 (0.0–2.0)	0.9 (0.0–2.9)	0.7 (0.0–2.0)

Normally distributed data mean ± SD; not normally distributed data and % values median and min/max; *SD* Standard deviation, *n* Number, *yrs* Years, *m* Meter, *kg* Kilogram, *% pred* % predicted, *ppb* Parts per billion

### Decrease of FEV_1_ after ECC and eNO before and after 4 weeks off sc-LCPUFA supplementation

**Fig. 2:**
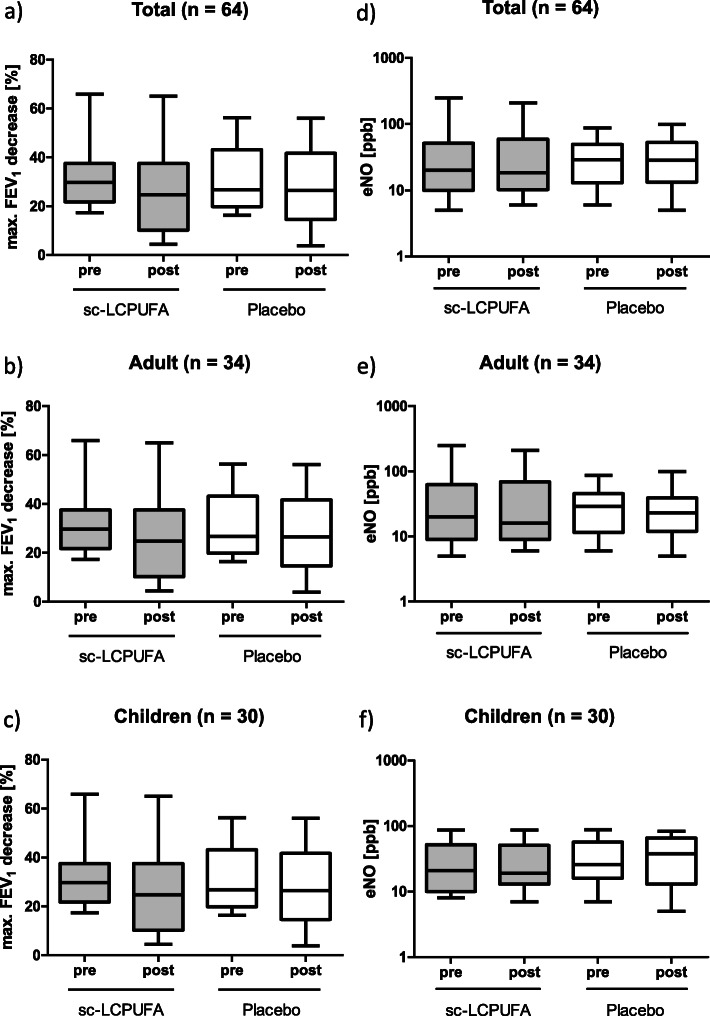
Max. FEV_1_ decrease and exhaled NO before and after sc-LCPUFA or placebo supplementation. Data for FEV_1_ decrease (**a**) Total *n* = 64; **b**) Adult *n* = 34; **c**) Children *n* = 30) and eNO (**d**), **e**), **f**)) were presented as median, 25%/75% percentile and min/max. The *P*-values were calculated with the Mann-Whitney-test. Results were considered as statistically significant when *P* < 0.05. None of the results were significant. The exact values for median and 25%/75% percentile are presented separately in Table 1 supplement

The maximal decrease of FEV_1_ after ECC showed no significant difference between the interventional and the placebo group before (31.7% ± 12.0 interventional; 31.1% ± 12.3 placebo; *P* = 0.90) and after 4 weeks of sc-LCPUFA or placebo supplementation (26.4% ± 17.7 interventional; 27.0% ±16.1 placebo; *P* = 0.86). The same results emerge in the subgroups for adult and children (Fig. 2 a-c, Table 1 supplement).

The eNO showed no significant difference between the interventional and the placebo group before (20.0 ppb (5.0–249.0) interventional; 29.0 ppb (6.0–88.0) placebo; *P* = 0.69) and after 4 weeks of PUFA or placebo supplementation (18.5 ppb (6.0–209.0) interventional; 28.5 ppb (5.0–99.0) placebo; *P* = 0.61). The same results emerge in the subgroups for adult (interventional group *n* = 17, placebo group n = 17) and children (interventional group *n* = 15, placebo group n = 15) (Fig. 2 d-f, Table 1 supplement).

These results were also confirmed by a multivariable linear mixed effect analysis where neither interventional vs. placebo, adult vs. children nor pairwise interactions with each other or time showed significant differences in the maximal decrease of FEV_1_ or eNO. Only for eNO, female patients had significantly lower eNO values (*P* < 0.001).

Subgroups with low eNO < 25 ppb and high eNO > 30 ppb were analyzed separately to revise the hypothesis whether the type of EIA with high eNO associated to more eosinophilic inflammation shows better response to sc-LCPUFA supplementation. The basis of the grouping was the eNO value in V2 (eNO < 25 ppb: interventional group *n* = 18, placebo group *n* = 14; eNO > 30 ppb: interventional group *n* = 12, placebo group n = 15). However, there were no significant differences in FEV_1_-decrease and eNO before and after treatment for both subgroups, neither in the direct comparisons nor in multivariable analysis (data not shown).

In addition, the effect of the sc-LCPUFA supplementation in patients with an increase of EPA in the blood cells of at least 0.25% (*n* = 28) and at least 0.5% (*n* = 21) were analysed as evidence for good compliance and good incorporation of EPA in the cell membrane. However, there were no significant differences in FEV_1_ decrease and eNO before and after treatment for both subgroups 0.25 and 0.5% respectively (data not shown).

### Side effects

The data of 58 patients (29 placebo group (90.6%), 29 interventional group (90.6%)) who completed their symptom diary on a regular basis were used for the calculations of side effects.

**Fig. 3:**
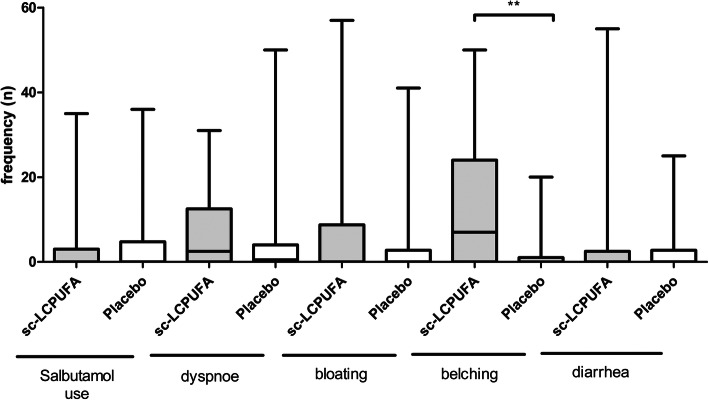
Frequency of the side effects during the sc-LCPUFA or placebo supplementation. The frequency of the side effects “salbutamol use”, “dyspnoe”, “bloating”, “belching” and “diarrhea” during the sc-LCPUFA or placebo supplementation are shown. Data were presented as median, 25%/75% percentile and min/max. The *P*-value was calculated with Mann-Whitney-test. Results were considered as statistically significant when *P* < 0.05. The frequency of the side effect “belching” showed a significant difference (*P* = 0.005) between the two groups, all the other side effects showed no significant difference between the interventional and placebo group

Patients in the interventional group complained significantly more often about “belching” [7 (0–50) vs. 0 (0–20) (*P* = 0.0048)]. Both, the gastrointestinal side effects “bloating” [0 (0–57) vs. 0 (0–41) (*P* = 0.18)] and “diarrhea” [0 (0–55) vs. 0 (0–25) (*P* = 0.8)] and the pulmonal complaints “use of salbutamol” [0 (0–35) vs. 0 (0–36) (*P* = 0.31)] or “dyspnoe” [2.5 (0–31) vs. 0.5 (0–50) (*P* = 0.28)] were not significantly different between interventional and placebo group (Fig. 3).

Furthermore, the side effects of two subgroups of the interventional group were analysed: those with an increase of EPA in the blood cells of at least 0.5% and those below. There were no significant differences in salbutamol use, dyspnoe, bloating, belching and diarrhoea for both subgroups (data not shown).

### Incorporation of sc-LCPUFA supplementation

**Fig. 4:**
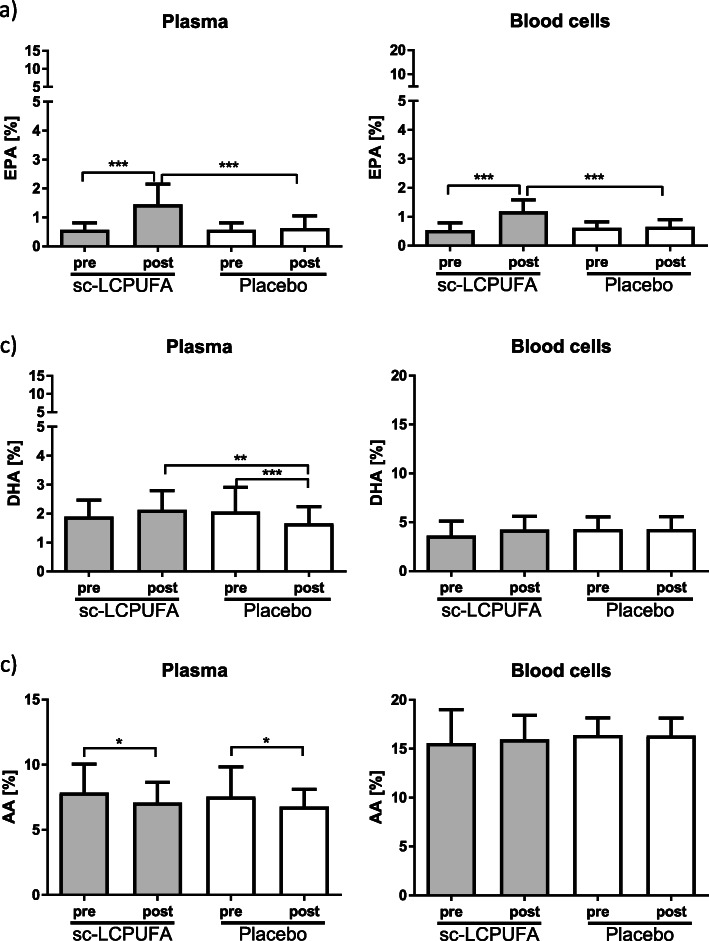
EPA, DHA and AA in plasma and blood cells before and after sc-LCPUFA or placebo supplementation. Data (n = 64) were presented as mean ± SD. The exact values for mean ± SD are presented separately in Table 2 in the Supplement. Intra-group and inter-group comparisons were calculated by One-Way ANOVA with post-hoc Bonferroni analysis. Results were considered as statistically significant when *p* < 0.05 (*** *P* < 0,001, ** *P* < 0.01, * *P* < 0.05)

Gas chromatographic analysis of plasma and blood cells of patients showed a significant increase of EPA at V3 compared to V2 in the investigational (*P* < 0.001), but not in the placebo group. However, PUFA supplementation did not increase DHA in plasma and blood cells of EIA patients (Fig. 4 a-c, Table 2 supplement). In comparison, placebo lowered DHA amounts in plasma of patients at V3 compared to V2 and patients who received the sc-LCPUFA.

Reduction of AA in plasma at V3 in both groups is probably due to a supplementation effect. However, there were no differences between the groups regarding the amount of AA. Results were the same in adults and children except for DHA which was significantly higher in plasma and blood cells in children (*P* < 0.05) of the investigational group at V3 compared to V2 (data not shown).

## Discussion

The aim of of this double-blind placebo-controlled study was to evaluate the effect of a sc-LCPUFA on EIA and airway inflammation in a large sample size of patients. The maximal decrease of FEV_1_ and eNO after the supplementation phase showed no significant difference between the interventional and the placebo group. Moreover, even a subgroup analysis in patients with EIA and high eNO were negative. PUFAs, such as eicosapentaenoic acid (EPA) and docosahexaenoic acid (DHA) have shown to decrease the production of pro-inflammatory eicosanoids, such as (cysteinyl)-leukotrienes, prostaglandins as well as pro-inflammatory cytokines by competitive inhibition of the arachidonic acid metabolism at cyclooxygenase-2 and 5- lipoxygenase enzymes [23, 24]. Moreover, EPA and DHA generate specialized pro-resolving mediators (SPMs) like protectins, resolvins, and maresins via several enzymatic reactions to counter-regulate airway eosinophilic inflammation and promote the resolution of inflammation in asthma [33, 34].

Thus the question arised why this study demonstrated no beneficial effect regarding lung function and eNO. Different reasons have to be discussed like the daily dose of LCPUFA, the proportion of EPA and DHA in the mixture, the provocation model used to induce an inflammatory response and the statistical power of the trial design.

Many studies showed a positive effect of n-3 LCPUFA supplementation in EIA with significantly higher doses > 1 g/d [10, 11, 13, 14]. A daily dose of 5.4 g n-3 PUFAs (3.2 g EPA and 2.0 g DHA) showed a significant reduction of eNO and FEV_1_ decrease in three different studies dealing with EIA [10, 11, 14]. In another study a dose of 3.1 g/d n-3 PUFAs and of 6.2 g/d n-3 PUFAs showed an equal reduction in eNO and FEV_1_ [13].

In contrast, there were many reports which found no clinical improvement after supplementation of high doses n-3 LCPUFA [16–20]. In a recent study supplementation with 3.0 g/d EPA and 3.0 g/d DHA did not show an effect on FEV_1_ decrease after eucapnic voluntary hyperpnea [19].

With increasing amount of n-3 LCPUFA supplementation, costs and gastrointestinal side effects increase, while compliance declines [13]. Therefore, it is interesting that other studies in EIA and asthmatic patients with low dose n-3 LCPUFA supplementation equivalent to this study again showed positive [12, 15] and negative [21, 22] results.

Supplementation with lipids of the New-Zealand green-lipped mussel (576 mg/d EPA, 384 mg/d DHA) for 3 weeks showed significant reduction of eNO and FEV_1_ decrease in EIA patients after dry air exercise challenge [12].

Summarized, there are positive and negative studies for both - high and low dose n-3 LCPUFA supplementation. Consequently, the total dose by itself cannot be the reason that no effect of sc-LCPUFA-supplementation could be demonstrated in this study. In addition, compliance of the patients was well controlled by keeping a diary and determination of the LCPUFA levels in plasma and blood cells. The latter is a well-established tool for dietary supplementation studies [24, 35]. However, as shown by Fig. 2 only EPA was significantly increased after supplementation, whereas DHA levels were unchanged. Thus, it is tempting to speculate that the DHA content of the sc-LCPUFA was maybe too low.

Current data suggest that EPA and DHA have distinct anti-inflammatory effects. EPA is endogenously converted into specialized pro-resolving mediators i.e. E-series resolvins, whereas DHA is converted into D-series resolvins, protectins and maresins [33]. Thus, it is important to well-balance a LCPUFA mixture, especially with a significant content of DHA [23, 24]. In mice DHA and Resolvin (Rv) D1 induce a phenotypic switch in macrophages from a pro-inflammatory towards an anti-inflammatory activation profile [34]. DHA was shown to be superior to EPA in respect of asthma prevention [36]. Furthermore, high concentrations of DHA were suggested to have a protective effect on lung function [37]. However, there is evidence that a combination of EPA and DHA exhibit the highest beneficial potential on chronic inflammation [10–15, 24].

Taken together, studies investigating a therapeutically effect of n-3 LCPUFA on bronchial asthma do not show consistent effects. The diverging study results may arise among others attributable by small study populations, different subtypes of asthma, different provocational methods and the different composition of the LCPUFA supplementation and the different duration of the supplementation period. In a review on this topic it was stated that negative results could be caused by methodological and statistical limitations [38]. In contrast to many other studies, this study used an ATS guideline conform exercise challenge. Thus the number of the patient cohort was well powered by previous results, mild and moderate asthmatics were tested as recommended and the subjects were well balanced (half adults, half children, mixed male and female). Consequently, the negative results of this trial cannot be attributed to methodological and statistical limitations.

However, it can be speculated that exercise per se is not an ideal model to induce a strong inflammatory response. In contrast, bronchial allergen provocation (BAP) in sensitised asthmatic individuals provokes a sustained late asthmatic reaction (LAR) in about 50–70% of individuals with asthma. The LAR may be attributable to a systemic and bronchial eosinophilic inflammation and correlated to exhaled NO. [39–42] In addition, patients with LAR show significantly higher eNO levels compared to patients with a negative BAP [40–43]. Thus, BAP seems to be more promising to study the possible anti-inflammatory effect of LCPUFA supplementation.

### Study limitations

There are some clinical limitations to this study. First, no data on the normal dietary habits of the patients (amount of weekly fish, meat, oils etc.) were gathered and no regulations for the diet during study participation were done. In addition, no eNO measurement 24 h after exercise was done to detect a possible late effect of the sc-LCPUFA supplementation.

## Conclusion

In conclusion, the sc-LCPUFA supplementation had no positive effect on EIA. Both, the maximal decrease of FEV_1_ and eNO as inflammatory marker were unchanged.

## Supplementary information

**Additional file 1: Table 1 Supplement.** Max. FEV_1_ decrease and eNO before and after sc-LCPUFA or placebo supplementation. % pred, % predicted; ppb, parts per billion.

**Additional file 2: Table 2 Supplement.** Fatty acid measurements in plasma and blood cells before and after sc-LCPUFA or placebo supplementation. Exact values for significant *P*-values: EPA Plasma: sc-LCPUFA pre – sc-LCPUFA post: < 0.0001, sc-LCPUFA post – Placebo post: > 0.0001. EPA Blood cells: sc-LCPUFA pre – sc-LCPUFA post: < 0.0001, sc-LCPUFA post – Placebo post: > 0.0001. DHA Plasma: sc-PUFA post – Placebo post: 0.004, Placebo pre – Placebo post: 0.0003. AA Plasma: sc-LCPUFA pre – sc-LCPUFA post: 0.037, Placebo pre – Placebo post: 0.014.

## Data Availability

The data from this study are available on request from the Department for Children and Adolescents, Division of Allergology, Pulmonology and Cystic fibrosis, Goethe-University, Frankfurt/Main, Germany.

## Abbreviations

ACQ: Asthma control questionnaire
APS: Aerosol provocation system
ATS: American Thoracic Society
BAP: Bronchial allergen provocation
BHR: Bronchial hyper-responsiveness
d: Day
DHA: Docosahexaenoic acid
ECC: Exercise challenge in a cold chamber
EIA: Exercise-induced asthma
EPA: Eicosapentaenoic acid
eNO: Exhaled nitric oxide
f: Female
FA: Fatty acid
FEV_1_: Forced expiratory volume in 1 s
FVC: Forced vital capacity
GLA: Gamma-Linolenic acid
ICS: Inhaled corticosteroid
LAR: Late asthmatic reaction
LCPUFA: Long-chain poly unsaturated fatty acid
m: Male
MCFA: Middle-chain fatty acids
mg: Milligrams
min: Minute
n: Number or Omega
PUFA: Poly unsaturated fatty acid
ppb: Parts per billion
Sc-LCPUFA: Special combination long-chain poly unsaturated fatty acid
SDA: Stearidonic acid
SPT: Skin prick test
SD: Standard deviation
SEM: Standard error of mean
V1/2/3: Visit 1/2/3

## References

[1] Bateman ED, Hurd SS, Barnes PJ, Bousquet J, Drazen JM, FitzGerald M (2008). Global strategy for asthma management and prevention: GINA executive summary. Eur Respir J.

[2] ElHalawani SM, Ly NT, Mahon RT, Amundson DE (2003). Exhaled nitric oxide as a predictor of exercise-induced bronchoconstriction. Chest.

[3] Lex C, Dymek S, Heying R, Kovacevic A, Kramm CM, Schuster A (2007). Value of surrogate tests to predict exercise-induced bronchoconstriction in atopic childhood asthma. Pediatr Pulmonol.

[4] Buchvald F, Hermansen MN, Nielsen KG, Bisgaard H (2005). Exhaled nitric oxide predicts exercise-induced bronchoconstriction in asthmatic school children. Chest.

[5] Grzelewski T, Grzelewska A, Majak P, Stelmach W, Kowalska A, Stelmach R (2012). Fractional exhaled nitric oxide (FeNO) may predict exercise-induced bronchoconstriction (EIB) in schoolchildren with atopic asthma. Nitric Oxide.

[6] Malmberg LP, Pelkonen AS, Mattila PS, Hammaren-Malmi S, Makela MJ (2009). Exhaled nitric oxide and exercise-induced bronchoconstriction in young wheezy children - interactions with atopy. Pediatr Allergy Immunol.

[7] Dreßler M, Salzmann-Manrique E, Zielen S, Schulze J (2018). Exhaled NO as a predictor of exercise-induced asthma in cold air. Nitric Oxide.

[8] Weiler JM, Brannan JD, Randolph CC, Hallstrand TS, Parsons J, Silvers W (2016). Exercise-induced bronchoconstriction update-2016. J Allergy Clin Immunol.

[9] Reddel HK, Bateman ED, Becker A, Boulet L, Cruz AA, Drazen JM (2015). A summary of the new GINA strategy: a roadmap to asthma control. Eur Respir J.

[10] Tecklenburg-Lund S, Mickleborough TD, Turner LA, Fly AD, Stager JM, Montgomery GS (2010). Randomized controlled trial of fish oil and montelukast and their combination on airway inflammation and hyperpnea-induced bronchoconstriction. PLoS One.

[11] Mickleborough TD, Murray RL, Ionescu AA, Lindley MR (2003). Fish oil supplementation reduces severity of exercise-induced bronchoconstriction in elite athletes. Am J Respir Crit Care Med.

[12] Mickleborough TD, Vaughn CL, Shei R, Davis EM, Wilhite DP (2013). Marine lipid fraction PCSO-524 (lyprinol/omega XL) of the New Zealand green lipped mussel attenuates hyperpnea-induced bronchoconstriction in asthma. Respir Med.

[13] Williams NC, Hunter KA, Shaw DE, Jackson KG, Sharpe GR, Johnson MA (2017). Comparable reductions in hyperpnoea-induced bronchoconstriction and markers of airway inflammation after supplementation with 6·2 and 3·1 g/d of long-chain n-3 PUFA in adults with asthma. Br J Nutr.

[14] Mickleborough TD, Lindley MR, Ionescu AA, Fly AD (2006). Protective effect of fish oil supplementation on exercise-induced bronchoconstriction in asthma. Chest.

[15] Nagakura T, Matsuda S, Shichijyo K, Sugimoto H, Hata K (2000). Dietary supplementation with fish oil rich in omega-3 polyunsaturated fatty acids in children with bronchial asthma. Eur Respir J.

[16] Brannan JD, Bood J, Alkhabaz A, Balgoma D, Otis J, Delin I (2015). The effect of omega-3 fatty acids on bronchial hyperresponsiveness, sputum eosinophilia, and mast cell mediators in asthma. Chest.

[17] Arm JP, Horton CE, Mencia-Huerta JM, House F, Eiser NM, Clark TJ (1988). Effect of dietary supplementation with fish oil lipids on mild asthma. Thorax.

[18] Kirsch CM, Payan DG, Wong MY, Dohlman JG, Blake VA, Petri MA (1988). Effect of eicosapentaenoic acid in asthma. Clin Allergy.

[19] Price OJ, Hull JH, Howatson G, Robson-Ansley P, Ansley L (2015). Vitamin D and omega-3 polyunsaturated fatty acid supplementation in athletes with exercise-induced bronchoconstriction: a pilot study. Expert Rev Respir Med.

[20] Covar R, Gleason M, Macomber B, Stewart L, Szefler P, Engelhardt K (2010). Impact of a novel nutritional formula on asthma control and biomarkers of allergic airway inflammation in children. Clin Exp Allergy.

[21] Moreira A, Moreira P, Delgado L, Fonseca J, Teixeira V, Padrão P (2007). Pilot study of the effects of n-3 polyunsaturated fatty acids on exhaled nitric oxide in patients with stable asthma. J Investig Allergol Clin Immunol.

[22] Hodge L, Salome CM, Hughes JM, Liu-Brennan D, Rimmer J, Allman M (1998). Effect of dietary intake of omega-3 and omega-6 fatty acids on severity of asthma in children. Eur Respir J.

[23] Beermann C, Neumann S, Fußbroich D, Zielen S, Schubert R (2016). Combinations of distinct long-chain polyunsaturated fatty acid species for improved dietary treatment against allergic bronchial asthma. Nutrition.

[24] Fussbroich D, Zimmermann K, Göpel A, Eickmeier O, Trischler J, Zielen S (2019). A specific combined long-chain polyunsaturated fatty acid supplementation reverses fatty acid profile alterations in a mouse model of chronic asthma. Lipids Health Dis.

[25] Recommendations for standardized procedures for the on-line and off-line measurement of exhaled lower respiratory nitric oxide and nasal nitric oxide in adults and children-1999. This official statement of the American Thoracic Society was adopted by the ATS Board of directors, July 1999. Am J Respir Crit Care Med. 1999;160(6):2104–17. 10.1164/ajrccm.160.6.ats8-99. 10.1164/ajrccm.160.6.ats8-99 10588636

[26] Parsons JP, Hallstrand TS, Mastronarde JG, Kaminsky DA, Rundell KW, Hull JH (2013). An official American Thoracic Society clinical practice guideline: exercise-induced bronchoconstriction. Am J Respir Crit Care Med.

[27] Dreßler M, Friedrich T, Lasowski N, Herrmann E, Zielen S, Schulze J (2019). Predictors and reproducibility of exercise-induced bronchoconstriction in cold air. BMC Pulm Med.

[28] Juniper EF, O'Byrne PM, Guyatt GH, Ferrie PJ, King DR (1999). Development and validation of a questionnaire to measure asthma control. Eur Respir J.

[29] Juniper EF, Bousquet J, Abetz L, Bateman ED (2006). Identifying 'well-controlled' and 'not well-controlled' asthma using the asthma control questionnaire. Respir Med.

[30] Jia CE, Zhang HP, Lv Y, Liang R, Jiang YQ, Powell H (2013). The asthma control test and asthma control questionnaire for assessing asthma control: systematic review and meta-analysis. J Allergy Clin Immunol.

[31] Juniper EF, Gruffydd-Jones K, Ward S, Svensson K (2010). Asthma control questionnaire in children: validation, measurement properties, interpretation. Eur Respir J.

[32] Miller MR, Hankinson J, Brusasco V, Burgos F, Casaburi R, Coates A (2005). Standardisation of spirometry. Eur Respir J.

[33] Serhan CN, Dalli J, Colas RA, Winkler JW, Chiang N (2015). Protectins and maresins: new pro-resolving families of mediators in acute inflammation and resolution bioactive metabolome. Biochim Biophys Acta.

[34] Titos E, Rius B, González-Périz A, López-Vicario C, Morán-Salvador E, Martínez-Clemente M (2011). Resolvin D1 and its precursor docosahexaenoic acid promote resolution of adipose tissue inflammation by eliciting macrophage polarization toward an M2-like phenotype. J Immunol.

[35] Kumar A, Mastana SS, Lindley MR (2016). N-3 fatty acids and asthma. Nutr Res Rev.

[36] Li J, Xun P, Zamora D, Sood A, Liu K, Daviglus M (2013). Intakes of long-chain omega-3 (n-3) PUFAs and fish in relation to incidence of asthma among American young adults: the CARDIA study. Am J Clin Nutr.

[37] Kompauer I, Demmelmair H, Koletzko B, Bolte G, Linseisen J, Heinrich J (2008). Association of fatty acids in serum phospholipids with lung function and bronchial hyperresponsiveness in adults. Eur J Epidemiol.

[38] Mickleborough TD, Rundell KW (2005). Dietary polyunsaturated fatty acids in asthma- and exercise-induced bronchoconstriction. Eur J Clin Nutr.

[39] de Monchy JG, Kauffman HF, Venge P, Koëter GH, Jansen HM, Sluiter HJ (1985). Bronchoalveolar eosinophilia during allergen-induced late asthmatic reactions. Am Rev Respir Dis.

[40] Kharitonov SA, O'Connor BJ, Evans DJ, Barnes PJ (1995). Allergen-induced late asthmatic reactions are associated with elevation of exhaled nitric oxide. Am J Respir Crit Care Med.

[41] Schulze J, Rosewich M, Dressler M, Riemer C, Rose MA, Zielen S (2012). Bronchial allergen challenge using the Medicaid dosimeter. Int Arch Allergy Immunol.

[42] Schulze J, Voss S, Zissler U, Rose MA, Zielen S, Schubert R (2012). Airway responses and inflammation in subjects with asthma after four days of repeated high-single-dose allergen challenge. Respir Res.

[43] Eickmeier O, Zissler UM, Wittschorek J, Unger F, Schmitt-Grohé S, Schubert R (2020). Clinical relevance of aspergillus fumigatus sensitization in cystic fibrosis. Clin Exp Allergy.

