# Supplementary material for: Oil supplementation with a special combination of n-3 and n-6 long-chain polyunsaturated fatty acids does not protect for exercise induced asthma: a double-blind placebo-controlled trial
Source: Lipids Health Dis. 2020 Jul 13;19:167. doi: 10.1186/s12944-020-01343-2 (PMC7359229; doi:10.1186/s12944-020-01343-2)
Supplement: Supplementary file 1 — Additional file 1: Table 1 Supplement. Max. FEV1 decrease and eNO before and after sc-LCPUFA or placebo supplementation. % pred, % predicted; ppb, parts per billion. [file 12944_2020_1343_MOESM1_ESM.docx]

**Table 1 Supplement: Max. FEV_1_ decrease and eNO before and after sc-LCPUFA or placebo supplementation**

|  | | **FEV_1_** | | | | **eNO** | | | |
| --- | --- | --- | --- | --- | --- | --- | --- | --- | --- |
|  |  | [% pred] | | | | [ppb] | | | |
|  |  | **sc-LCPUFA** | | **Placebo** | | **sc-LCPUFA** | | **Placebo** | |
|  |  | **pre** | **post** | **pre** | **post** | **pre** | **post** | **pre** | **post** |
| **Total** | **Median** | **29.8** | **24.8** | **26.8** | **26.5** | **20.0** | **18.5** | **29.0** | **28.5** |
|  | 25% percentile | 21.8 | 10.3 | 19.9 | 14.6 | 10.0 | 10.3 | 13.0 | 13.3 |
|  | 75% percentile | 37.6 | 37.6 | 43.2 | 41.7 | 51.3 | 59.3 | 49.3 | 52.8 |
| **Adult** | **Median** | **29.2** | **26.2** | **23.0** | **26.4** | **20.0** | **16.0** | **29.0** | **23.0** |
|  | 25% percentile | 21.8 | 10.9 | 20.1 | 15.2 | 9.0 | 9.0 | 11.5 | 12.0 |
|  | 75% percentile | 35.0 | 33.7 | 38.0 | 32.0 | 62.5 | 69.0 | 45.5 | 39.0 |
| **Children** | **Median** | **30.6** | **20.8** | **32.0** | **26.7** | **21.0** | **19.0** | **26.0** | **38.0** |
|  | 25% percentile | 21.4 | 7.8 | 19.2 | 9.4 | 10.0 | 13.0 | 16.0 | 13.0 |
|  | 75% percentile | 45.6 | 44.1 | 44.8 | 48.2 | 52.0 | 51.0 | 57.0 | 66.0 |

% pred, % predicted; ppb, parts per billion.
