# Supplementary material for: Oil supplementation with a special combination of n-3 and n-6 long-chain polyunsaturated fatty acids does not protect for exercise induced asthma: a double-blind placebo-controlled trial
Source: Lipids Health Dis. 2020 Jul 13;19:167. doi: 10.1186/s12944-020-01343-2 (PMC7359229; doi:10.1186/s12944-020-01343-2)
Supplement: Supplementary file 2 — Additional file 2: Table 2 Supplement. Fatty acid measurements in plasma and blood cells before and after sc-LCPUFA or placebo supplementation. Exact values for significant P-values: EPA Plasma: sc-LCPUFA pre – sc-LCPUFA post: < 0.0001, sc-LCPUFA post – Placebo post: > 0.0001. EPA Blood cells: sc-LCPUFA pre – sc-LCPUFA post: < 0.0001, sc-LCPUFA post – Placebo post: > 0.0001. DHA Plasma: sc-PUFA post – Placebo post: 0.004, Placebo pre – Placebo post: 0.0003. AA Plasma: sc-LCPUFA pre – sc-LCPUFA post: 0.037, Placebo pre – Placebo post: 0.014. [file 12944_2020_1343_MOESM2_ESM.docx]

**Table 2 Supplement:** **Fatty acid measurements in plasma and blood cells before and after sc-LCPUFA or placebo supplementation**

|  | | **plasma** | | | | **blood cells** | | | |
| --- | --- | --- | --- | --- | --- | --- | --- | --- | --- |
|  |  | **sc-LCPUFA** | | **Placebo** | | **sc-LCPUFA** | | **Placebo** | |
|  |  | pre | post | pre | post | pre | post | pre | post |
| **EPA [%]** | mean | 0.57 | 1.45 | 0.57 | 0.63 | 0.53 | 1.19 | 0.62 | 0.65 |
|  | SD | 0.23 | 0.7 | 0.24 | 0.43 | 0.26 | 0.40 | 0.20 | 0.25 |
| **DHA [%]** | mean | 1.90 | 2.13 | 2.07 | 1.66 | 3.63 | 4.24 | 4.29 | 4.29 |
|  | SD | 0.57 | 0.67 | 0.84 | 0.58 | 1.50 | 1.36 | 1.27 | 1.29 |
| **AA [%]** | mean | 7.86 | 7.10 | 7.56 | 6.78 | 15.57 | 15.98 | 16.41 | 16.34 |
|  | SD | 2.20 | 1.55 | 2.27 | 1.33 | 3.44 | 2.44 | 1.76 | 1.80 |

Exact values for significant p-values:
EPA Plasma: sc-LCPUFA pre – sc-LCPUFA post: < 0.0001, sc-LCPUFA post – Placebo post: > 0.0001
EPA Blood cells: sc-LCPUFA pre – sc-LCPUFA post: < 0.0001, sc-LCPUFA post – Placebo post: > 0.0001
DHA Plasma: sc-PUFA post – Placebo post:0.004, Placebo pre – Placebo post: 0.0003
AA Plasma: sc-LCPUFA pre – sc-LCPUFA post: 0.037, Placebo pre – Placebo post: 0.014
